# Isolation of *Mycobacterium arupense* from pleural effusion: culprit or not?

**DOI:** 10.1186/s12879-018-3136-3

**Published:** 2018-05-15

**Authors:** Xian Zhou, Qiaoling Ruan, Weimin Jiang, Xinyu Wang, Yuan Jiang, Shenglei Yu, Yu Xu, Jing Li, Yangyi Zhang, Wenhong Zhang, Yuekai Hu

**Affiliations:** 1 Department of Infectious Diseases, Huashan Hospital, Fudan University, 12 M. Wulumuqi Road, Shanghai, 200040 China; 2 Department of Laboratory Medicine, Huashan Hospital, Fudan University, Shanghai, China; 3 Shanghai Municipal Center for Disease Control and Prevention, Shanghai, China

**Keywords:** *Mycobacterium arupense*, Nontuberculous mycobacterium, Pleural effusion, Chylothorax

## Abstract

**Background:**

*Mycobacterium arupense*, first identified in 2006, is a slow-growing nontuberculous mycobacterium (NTM) and an emerging cause of tenosynovitis, potentially associated with immunosuppression. However, unlike the diagnostic value of its isolation from osteoarticular specimens, the significance of detecting *M. arupense* in respiratory specimens is not yet clear.

**Case presentation:**

To our knowledge, we, for the first time, described the identification of *M. arupense* from the pleural effusion of an immunocompetent patient, who presented with fever and chylothorax. The symptoms resolved with doxycycline treatment for 45 days and a low-fat, high-protein diet. Follow-up at 14 months showed no relapse.

**Conclusions:**

Because the patient fully recovered without combined anti-NTM treatment, we did not consider *M. arupense* the etiological cause in this case. This indicates that *M. arupense* detected in pleural effusion is not necessarily a causative agent and careful interpretation is needed in terms of its clinical relevance.

**Electronic supplementary material:**

The online version of this article (10.1186/s12879-018-3136-3) contains supplementary material, which is available to authorized users.

## Background

*Mycobacterium arupense*, first identified in 2006, is a slow-growing nontuberculous mycobacterium (NTM) belonging to *M. terrae* complex [1]. Although it is widespread in the environment, *M. arupense* is considered to cause NTM infection rarely, most commonly affecting patients with immunodeficiency or history of injury [2]. Here, we describe the identification of this species in the pleural effusion of an immunocompetent patient.

## Case presentation

**Fig. 1:**
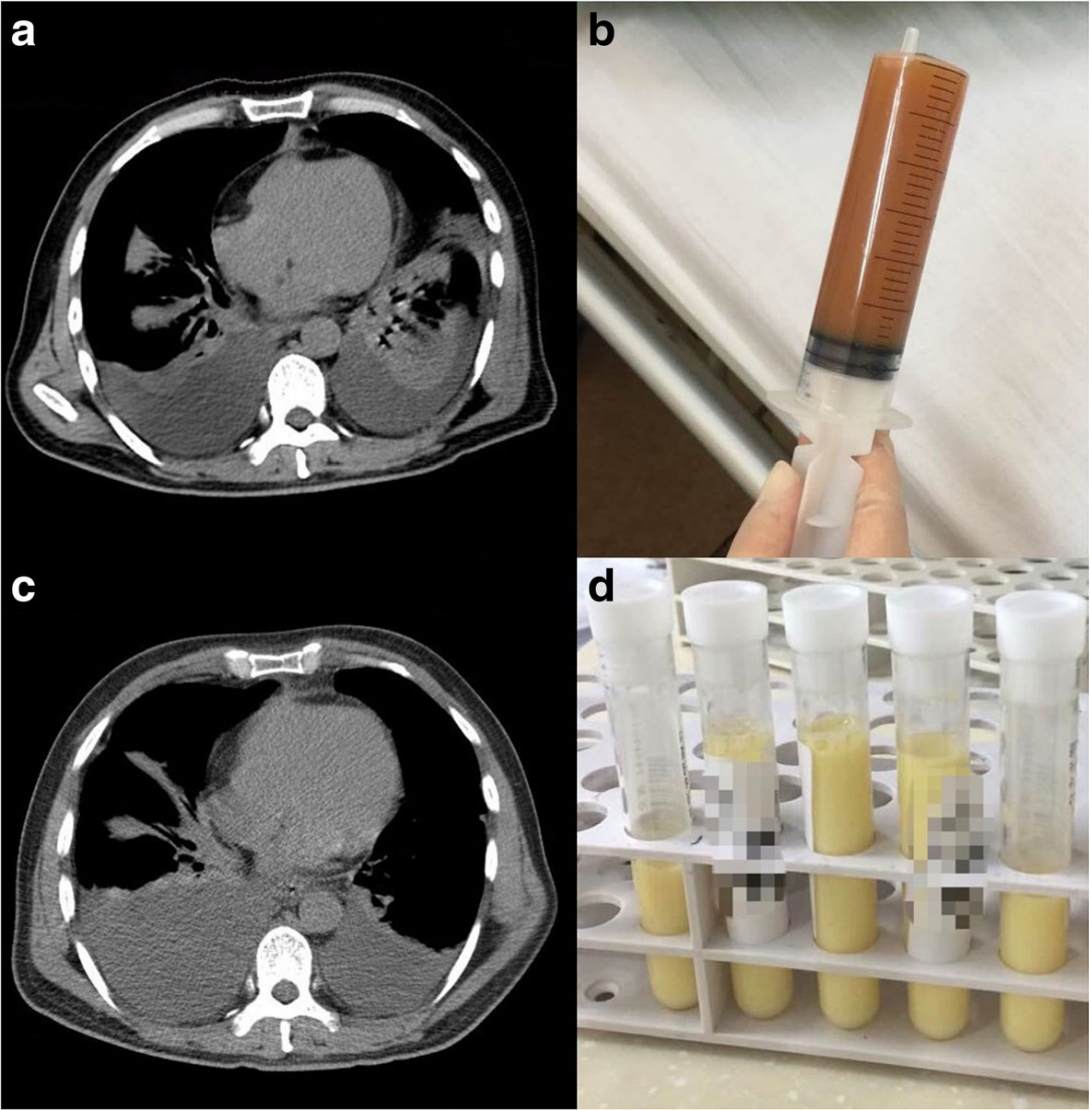
Pleural effusion of a 50-year-old male farmer. **a** Computed tomography revealed bilateral pleural effusion about a week after the onset of symptom. **b** Pleural effusion from the first thoracentesis. **c** Computed tomography showed that pleural effusion continued. **d** Pleural effusion from the second thoracentesis revealed chylothorax

A previously healthy, 50-year-old farmer was admitted because of fever and generalized edema. Two weeks prior to hospitalization, fever (up to 39.8 °C), chills, and sore throat developed. Physical examination at a local hospital did not reveal significant findings, but only an oral ulcer. A week later, pharyngalgia worsened and shortness of breath developed. Laryngoscopy showed severe edema of the epiglottis and arytenoid cartilage, and chest computed tomography revealed bilateral pleural effusion (Fig. 1a). Diagnosed as acute laryngitis, 10 mg dexamethasone was administered intravenously daily for 4 days. The patient’s edema of the epiglottis and fever improved, but the edema extended to the whole body. After discontinuation of dexamethasone, the patient experienced a fever of 38 °C. Therefore, he was transferred to our hospital.

On physical examination, the patient showed systemic edema, most severe in the scrotum and both the lower extremities. The white blood cell (WBC) count was 7.03 × 10^9^/L, with 75.2% neutrophils. Serum albumin, pro-BNP, and C-reactive protein levels were 27.9 g/L, 55.7 pg/mL, and 6.3 mg/L, respectively. The patient had no diabetes mellitus. The serum immunoglobulin levels were within normal ranges. HIV was negative with CD4 counts of 531/mm^3^. Pleural fluid (Fig. 1b) analysis showed: total protein, 35.5 g/L; lactate dehydrogenase, 126 U/L; red blood cell, 120 × 10^6^/L; WBC, 160 × 10^6^/L with 2% neutrophils, 35% lymphocytes, and 63% macrophages. The fever was persistent even after the empirical meropenem treatment. Repeated blood cultures were negative for bacteria and fungi. As the patient reported close contact with cats and dogs, doxycycline was added in the treatment and the patient’s temperature returned to normal immediately. However, the edema and bilateral pleural effusion continued despite the diuretics and albumin supportive treatment. The second thoracentesis confirmed chylothorax (Fig. 1c-d), with triglyceride levels at 1389 mg/dL. Malignant cells were not found in the pleural effusion. Thick blood film smears for microfilariae and vascular ultrasound for thrombus showed negative results. Therefore, we decreased the patient’s intravenous fluid supplement and the symptoms were relieved with a low-fat, high-protein diet.

*Mycobacterium* species from the first pleural effusion specimen were cultured successfully on the Löwenstein-Jensen medium after 23 days. The culture smear was positive for acid-fast bacilli. Following inoculation to MGIT 960 liquid system and the solid Löwenstein-Jensen medium were both positive for mycobacteria. The partial 16S rRNA gene sequence of the isolate (482 bp) was 99.17% similar to that of the *M. arupense* strain AR30097. The susceptibility testing of the isolates for capreomycin and moxifloxacin yielded minimum inhibitory concentrations of 64 μg/mL and > 16 μg/mL, respectively. By the time *M. arupense* was confirmed, the patient recovered and refused the proposal of anti-NTM treatment. Follow-up at 3 months after discharge showed no pleural effusion, and follow-up at 14 months showed no relapse.

## Discussion and conclusions

Isolation of *M. arupense* has been reported from at least 118 clinical samples [2]. These strains were detected mainly in two types of specimens: 26 in tissue specimens and 92 in respiratory specimens (Additional file 1: Table S1). Only one positive blood culture report was obtained from an acquired immune deficiency syndrome (AIDS) patient who was diagnosed with disseminated *M. arupense* infection [3]. Ten out of 26 strains from tissue specimens were reported in a 2016 study by Vasireddy et al. [4]. They identified *M. arupense* by sequencing the 26 clinical isolates of tenosynovitis or osteomyelitis previously diagnosed as *M. terrae* and *M. nonchromogenicum* from 1984 to 2014 in the U.S. Patients with *M. arupense* osteoarticular infection usually had a history of an injury or corticosteroid use. Although there is no standard well-established treatment for *M. arupense* infection, these patients showed favorable prognosis after surgery and 6–14 months of combined antimicrobial treatment.

Unlike the diagnostic value of its isolation in osteoarticular specimens, the significance of detecting *M. arupense* in respiratory specimens is not yet clear. Since the first *M. arupense* pulmonary infection in a patient with kidney neoplasm reported in 2010 [5], *M. arupense* has only been reported as a pathogen causing pulmonary infection in 2 other patients, both with AIDS [3, 6] (Additional file 1: Table S1). Fifty-three of 92 *M. arupense* isolates from respiratory specimens were reported in a study retrospectively reviewing 53 cancer patients with positive cultures of *M. arupense* from 2007 to 2014 [7]. The outcomes of 13 treated patients and 40 untreated ones did not differ significantly. These results suggest that *M. arupense* can cause pulmonary infection in immunocompromised patients, but is usually a “bystander” in respiratory infections.

This is the first case report, to our knowledge, on the isolation of *M. arupense* from pleural effusion. There were isolations reported previously [1, 7], but with minimal clinical information. Although the possibility of contamination is low, we did not consider *M. arupense* as an etiological cause in our case for the following three reasons. First, NTM that was isolated from the pleural effusion should not be considered etiologic unless there is evidence of NTM infection in other tissues [8]. Second, the patient recovered with doxycycline treatment for 45 days. Although we did not perform a drug susceptibility test to doxycycline of the isolate due to limited resources, drug susceptibility testing of 40 strains of *M. arupense* reported by Beam et al. found that only 5 out of 23 (21.7%) isolates were susceptible to doxycycline [9]. There is no report of doxycycline mono treatment for NTM cases. Lastly, NTM related chylothorax has been reported in only one AIDS patient [10], but not in immunocompetent patients. Although these observations are based on a single patient and lack a control group or other studies for comparison, this case indicates that *M. arupense* found in pleural effusion is not necessarily a causative agent and careful interpretation is required to pinpoint its clinical relevance.

## Additional file

Additional file 1: **Table S1.** Summary of pulmonary isolates of *M. arupense*. A table of cases with pulmonary isolates of *M. arupense* over the years with clinical history. (DOCX 20 kb)

## Abbreviations

AIDS: Acquired immune deficiency syndrome
NTM: Nontuberculous mycobacterium
WBC: White blood cell
