# Supplementary material for: Isolation of Mycobacterium arupense from pleural effusion: culprit or not?
Source: BMC Infect Dis. 2018 May 15;18:221. doi: 10.1186/s12879-018-3136-3 (PMC5952631; doi:10.1186/s12879-018-3136-3)
Supplement: Supplementary file 1 — Table S1. Summary of pulmonary isolates of M. arupense. A table of cases with pulmonary isolates of M. arupense over the years with clinical history. (DOCX 20 kb) [file 12879_2018_3136_MOESM1_ESM.docx]

**Table S1. Summary of pulmonary isolates of *M. arupense***

| Reference | No. of cases | Comordities | Specimen | Detailed clinical history | Anti-NTM treatment | Outcome |
| --- | --- | --- | --- | --- | --- | --- |
| Masaki T, et al. 2006[1] | n=1 | NA | Sputum | A 60-year-old Japanese man with abnormal shadow by indirect X-ray. | NA | NA |
| Neonakis et al. 2010[2] | n=1 | Undiagnosed kidney neoplasm | Sputum | A 62-year-old man with one-month history of recurrent fever, dyspnea and haemoptysis. | Levofloxacin, piperacillin/tazobactam and clarithromycin | Improvement |
| Slany M, et al. 2010[3] | n=3 | Diabetes mellitus | Sputum(tiwce) | A 55-year-old woman with respiratory complications, increased temperature and fatigue. Abnormalities in the X-ray. | No | Improvement |
|  |  | No | Sputum | A 66-year-old male with an intensive cough giving rise to yellow or pink Sputum, increased temperature and fatigue. Radiography showed a round shadow in the right lung. | No | Partial regression |
|  |  | No | Sputum (3 times) | A 77-year-old woman with no health complications and a clean X-ray screen. | No | Well |
| Heidarieh et al. 2013[4] | n=1 | HIV/AIDS | Sputum (at lease twice)and BAL | A 55-year-old Iranian man suffering from chronic pulmonary disease. Nornam chest X-ray. | Clarithromycin, ethambutol, rifabutin | Improvement |
| Beam et al. 2014[5] | n=32 | NA | Sputum(n=23), BAL/BW(n=9), | NA | NA | NA |
| Al Hamal et al. 2016[6] | n=53 | Cancer | BAL(n=9), Sputum(n=4) | NA | Yes (Details unavailable) | Improvement(n=7, worsening(n=3), mixed(n=1) |
|  |  | Cancer | BAL(n=19), Sputum(n=20), Pleural fluid(n=1) | NA | No | Improvement(n=20), worsening(n=7), mixed(n=1), no change(n=4) |
| Verghese et al. 2017[7] | n=1 | HL, COPD, and HIV | BAL | A 30-year-old Saudi woman | Clarithromycin, ethambutol, and rifampin | NA |

NA, not available; BAL, bronchoalveolar lavage; BW, bronchoalveolar washing; HL, Hodgkin lymphoma; COPD, chronic obstructive pulmonary disease

**References:**

1. Masaki T, Ohkusu K, Hata H, Fujiwara N, Iihara H, Yamada Noda M, Nhung PH, Hayashi M, Asano Y, Kawamura Y: **Mycobacterium kumamotonense sp. nov. recovered from clinical specimen and the first isolation report of Mycobacterium arupense in Japan: novel slowly growing, nonchromogenic clinical isolates related to Mycobacterium terrae complex**. *MICROBIOL IMMUNOL* 2006, **50**(11):889-897.

2. Neonakis IK, Gitti Z, Kontos F, Baritaki S, Petinaki E, Baritaki M, Liakou V, Zerva L, Spandidos DA: **Mycobacterium arupense pulmonary infection: antibiotic resistance and restriction fragment length polymorphism analysis**. *INDIAN J MED MICROBI* 2010, **28**(2):173.

3. Slany M, Svobodova J, Ettlova A, Slana I, Mrlik V, Pavlik I: **Mycobacterium arupense among the isolates of non-tuberculous mycobacteria from human, animal and environmental samples**. *VET MED-CZECH* 2010, **55**(8):369-376.

4. Heidarieh P, Hashemi-Shahraki A, Khosravi AD, Zaker-Boustanabad S, Shojaei H, Feizabadi MM: **Mycobacterium arupense infection in HIV-infected patients from Iran**. *INT J STD AIDS* 2013, **24**(6):485-487.

5. Beam E, Vasoo S, Simner PJ, Rizzo M, Mason EL, Walker RC, Deml SM, Brown-Elliott BA, Wallace RJ, Wengenack NL *et al*: **Mycobacterium arupense Flexor Tenosynovitis: Case Report and Review of Antimicrobial Susceptibility Profiles for 40 Clinical Isolates**. *J CLIN MICROBIOL* 2014, **52**(7):2706-2708.

6. Al Hamal Z, Jordan M, Hachem RY, Alawami HM, Alburki AM, Yousif A, Deshmukh P, Jiang Y, Chaftari A, Raad II: **Mycobacterium arupense in Cancer Patients**. *MEDICINE* 2016, **95**(14):e2691.

7. Varghese B, Enani M, Shoukri M, AlThawadi S, AlJohani S, Al-Hajoj S: **Emergence of Rare Species of Nontuberculous Mycobacteria as Potential Pathogens in Saudi Arabian Clinical Setting**. *PLOS NEGLECT TROP D* 2017, **11**(1):e5288.
